# Investigation of sphingosine kinase 1 in interferon responses during dengue virus infection

**DOI:** 10.1038/cti.2017.32

**Published:** 2017-07-21

**Authors:** Amanda L Aloia, Julie K Calvert, Jennifer N Clarke, Lorena T Davies, Karla J Helbig, Stuart M Pitson, Jillian M Carr

**Affiliations:** 1 Department of Microbiology and Infectious Diseases, School of Medicine, Flinders Medical Centre, Flinders University, Adelaide, South Australia, Australia; 2 Centre for Cancer Biology, University of South Australia and SA Pathology, Adelaide, South Australia, Australia; 3 Department of Physiology, Anatomy and Microbiology, La Trobe University, Melbourne, Victoria, Australia

## Abstract

Dengue virus (DENV) regulates sphingosine kinase (SK)-1 activity and chemical inhibition of SK1 reduces DENV infection. In primary murine embryonic fibroblasts (pMEFs) lacking SK1 however, DENV infection is enhanced and this is associated with induction of normal levels of interferon beta (IFN-β) but reduced levels of IFN-stimulated genes (ISGs). We have further investigated this link between SK1 and type I IFN responses. DENV infection downregulates cell-surface IFN-alpha receptor (IFNAR)1 in both wild-type (WT) and SK1^−^^/−^ pMEF, but, consistent with poor ISG responses, shows reduced induction of phosphorylated (p)-STAT1 and key IFN regulatory factors (IRF)1 and −7 in SK1^−/−^ pMEF. Direct IFN stimulation induced ISGs (viperin, IFIT1), CXCL10, IRF1 and −7 and p-STAT1. Responses, however, were significantly reduced in SK1^−/−^ pMEF, except for IFN-stimulated CXCL10 and IRF7. Poor IFN responses in SK1^−/−^ pMEF were associated with a small reduction in basal cell-surface IFNAR1 and IRF1 mRNA in uninfected SK1^−/−^ compared with WT pMEF. In contrast, treatment of cells with the SK1 inhibitor, SK1-I or expression of an inhibitory SK1 short hairpin RNA (shRNA), both of which reduce DENV infection, does not alter basal IRF1 mRNA or affect type I IFN stimulation of p-STAT1. Thus, cells genetically lacking SK1 can induce many responses normally following DENV infection, but have adaptive changes in IFNAR1 and IRF1 that compromise DENV-induced type I IFN responses. This suggests a biological link between SK1 and IFN-stimulated pathways. Other approaches to reduce SK1 activity, however, do not influence these important antiviral pathways but reduce infection and may be useful antiviral strategies.

Dengue virus (DENV) is a positive-strand RNA virus of the *flavivirus* family that is estimated to infect around 100 million people each year, resulting in ~500 000 hospitalisations and 25 000 deaths.^1^ With the geographic range of the mosquitoes responsible for spreading DENV increasing, DENV infections are likely to increase.^2^ A new DENV vaccine has recently been licenced in some countries but no direct-acting antiviral treatments are available. The progression of DENV disease has been suggested to be an immunopathogenesis, whereby the immune system is dysregulated. Thus, there has been much focus on the roles of innate and interferon (IFN)-driven responses during DENV infection^3, 4, 5^ and the association of this with the induction of a pathogenic cytokine response.^6, 7, 8^

*In vivo*, DENV replication primarily occurs in monocytes, macrophages and dendritic cells.^9, 10^ Following infection DENV RNA is detected by pattern recognition receptors such as toll-like receptors and retinoic acid-inducible gene 1 (RIG-I).^5, 11^ This subsequently elicits an antiviral response via pathways initiated by intracellular factors including IFN regulatory factors (IRFs). These IRFs have an important role in stimulating transcription and production of factors such as IFNs, tumour necrosis factor-α (TNF-α) and interleukins. The major ‘canonical’ type I IFN pathway is stimulated by IFN-α/β binding to a heterodimeric complex of IFN-α/β receptor (IFNAR)-1 and IFNAR2^12^ and subsequently activating JAK/signal transducer and activator of transcription (STAT)-1 and STAT2 by a cascade of phosphorylation events. Phosphorylated STAT1 and STAT2 interact with IRF9 to form the ISGF3 complex that binds to IFN-sensitive response elements in IFN-stimulated genes (ISGs) such as key antivirals: viperin and IFN Induced Protein With Tetratricopeptide Repeats 1 (IFIT1), and also IRFs themselves, thus creating a positive feedback loop between IRFs, IFN and ISGs.^13, 14^ IRF3 and IRF7 are traditionally thought to respond to RNA viruses and IRF7 has been demonstrated to have a role in protective responses against DENV infection.^15^ While these are generalities in cellular responses to viral infection, the induction of these antiviral responses are coordinated and distinctly regulated by a growing number of complex interacting pathways.

One new cellular factor that we have identified to influence the IFN response that is induced following DENV infection is sphingosine kinase1 (SK1). SK1 is the major isoform of SK in most cells and tissues and functions in the sphingolipid metabolic pathway to convert sphingosine to sphingosine-1-phosphate (S1P).^16, 17^ S1P can function intracellularly and can also be exported extracellularly to act via a family of five G-protein-coupled receptors (S1PR1–5) to elicit growth promoting and immunomodulatory functions.^16, 17, 18, 19^ A few studies have linked the SK1/S1P axis to intracellular cytokine signalling pathways. Work from our laboratory and others have shown that TNF-α stimulation has reduced capacity to activate NFκB when SK1 is absent or reduced.^20, 21, 22^ Later work by others has described an association of SK1/S1P with TNF receptor-associated factor 2 to activate nuclear factor κB (NFκB) following TNF-α stimulation.^23^ More recently however, this has been questioned with SK1 deemed not essential for TNF-α-induced activation of NFκB and inflammatory responses in keratinocytes^24^ or macrophages.^25^ In addition, interleukin-1-stimulated and IRF1-mediated induction of CXCL10 and CCL5 requires phosphorylated SK1 that complexes with TNF receptor-associated factor 6, S1P and cIAP2 to promote IRF1 ubiquitination. The ubiquitinated IRF1 can be activated by phosphorylation and can translocate to the nucleus to interact with NFκB and induce CXCL10 and CCL5 expression.^26^

In response to infection, overexpression of SK1 reduces the capacity of influenza A virus (IAV) to induce p-STAT1, and these authors have proposed a role for SK1 in negatively regulating IFN signalling.^27, 28^ Our previous work has defined changes in SK1 activity during DENV infection^20, 29^ but discrepant effects of modulating SK1 on DENV infection; overexpression of SK1 has no effect, chemical inhibition of SK1 or infection of an immortalised SK1^−/−^ murine embryonic fibroblast line reduces infection, but infection of multiple isolates of SK1^−/−^ primary (p)MEF are enhanced.^30^ Studying the induction of ISGs is confounded in models such as immortalised SK1^−/−^ murine embryonic fibroblast and SK1 drug inhibition, since DENV replication is concurrently reduced. In the latter SK1^−/−^ primary murine embryonic fibroblast (pMEF) model, however, we observed reduced mRNA levels of the ISGs IFIT1, viperin and the ISG chemokine CXCL10 following DENV infection, despite higher replication and normal-higher IFN-β levels than wild-type (WT) pMEF.^30^ This implies that a lack of SK1 affects events post-IFN-β production and/or IFN-β-independent pathways that culminate in the induction of IFIT1, viperin and CXCL10 in response to DENV infection.

Here we have further defined the responses of cells with reduced SK1 to DENV infection and type I IFN stimulation. We show that cells genetically lacking SK1 show some elements of normal responses to DENV infection but have reduced ability to induce key ISGs following DENV infection or IFN-β stimulation. This is associated with a small decrease in uninfected cells of cell-surface IFNAR1 and IRF1 (mRNA)—both major mediators and enhancers of ISG expression—as well as reduced ability to induce Y701-p-STAT1—an important post-receptor signalling pathway for type I IFN activation of ISGs. These changes in basal IRF1 and p-STAT1 are not evident in uninfected cells treated either chemically or with a short hairpin RNA (shRNA) to reduce SK1. Our results suggest that the difference in ability of DENV to infect cells lacking SK1 (SK1^−/−^), where infection is enhanced, compared with cells with reduced SK1 (SK1-I; SK1 shRNA), where infection is reduced, is likely due to adaptive changes in key components of the IFN response in SK1^−/−^ cells. These findings have important implications for interpretation of genetic manipulations of SK1 in relation to IFN and antiviral responses and the potential for use of SK1 inhibitors as therapeutics in an infectious setting.

## Results

### Some responses to DENV infection are similar in both WT and SK1^−/−^ MEF, but induction of key IFN responses are reduced in SK1^−/−^ pMEF

**Figure 1:**
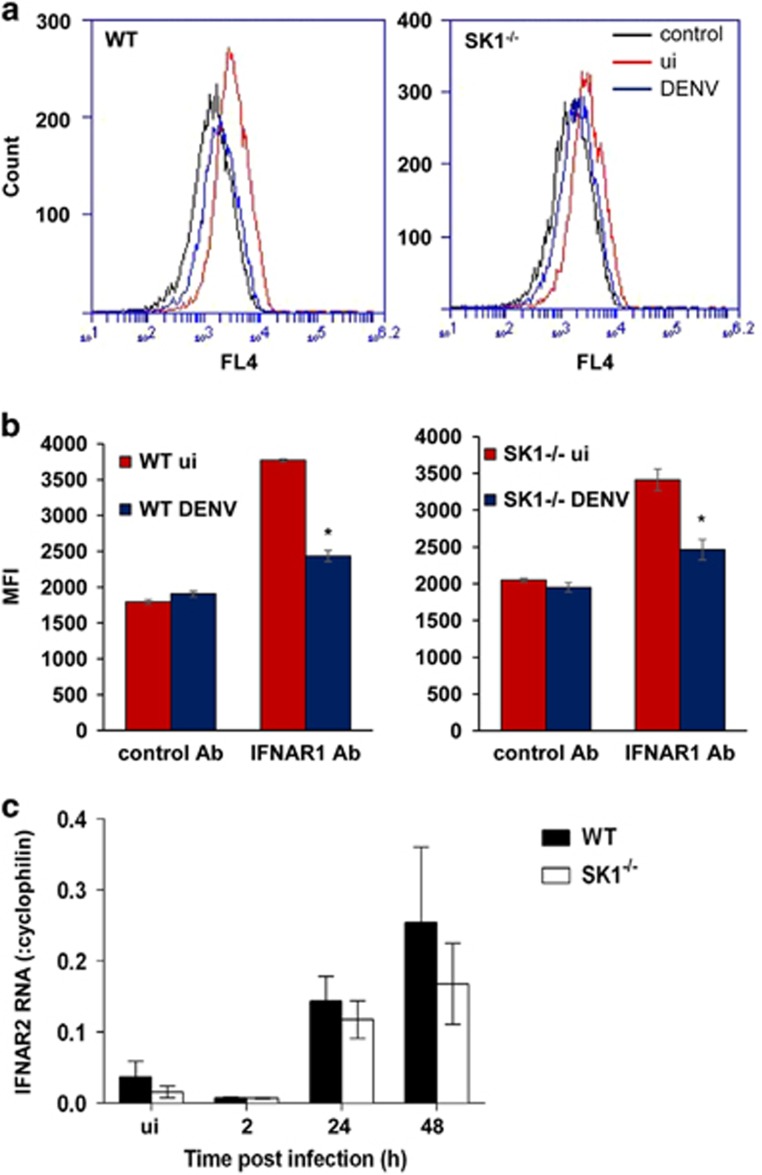
Cell-surface IFNAR1 is reduced and IFNAR2 mRNA is increased in both DENV-infected WT and SK1^−/−^ pMEF. WT and SK1^−/−^ pMEF were left uninfected or infected with DENV, as indicated directly on figures. (**a**) At 24 h pi cells were harvested and stained using APC-conjugated IFNAR1 or isotype control antibodies. Cells were fixed and subjected to flow cytometry. Representative histograms are shown; (**b**) as in **a** with av±s.e.m. of mean florescent intensity (MFI) from replicate (*n*=3) flow analysis from *n*=2 independent pMEF preparations; (**c**) RNA was extracted at the indicated time point pi and subjected to RT-PCR for IFNAR2. Results were normalised against cellular cyclophilin and relative RNA level determined by ΔCt method. Data represent av±s.e.m. from *n*=2 RT-PCR values from *n*=3 independent pMEF preparations and infection experiments. Representative replication profile of infectious DENV release is shown in Figure 2a. *P<0.05, Student's unpaired *t*-test.

Our prior data have suggested that the induction of ISGs are reduced in DENV-infected SK1^−/−^ pMEF,^30^ so to assess the type I IFN signalling pathways in SK1^−/−^ pMEF following DENV infection we measured IFNAR1 protein by flow cytometry and IFNAR2 mRNA by reverse transcriptase polymerase chain reaction (RT-PCR). Cell-surface IFNAR1 was significantly reduced at 24 h pi in both DENV-infected WT and SK1^−/−^ pMEF (Figures 1a and b). In contrast, at the mRNA level, IFNAR2 was significantly induced following DENV infection, but was not significantly different in WT compared to SK1^−/−^ pMEF (Figure 1c). This demonstrates similar responses of the type I IFN receptor to DENV infection in WT and SK1^−/−^ pMEF.

**Figure 2:**
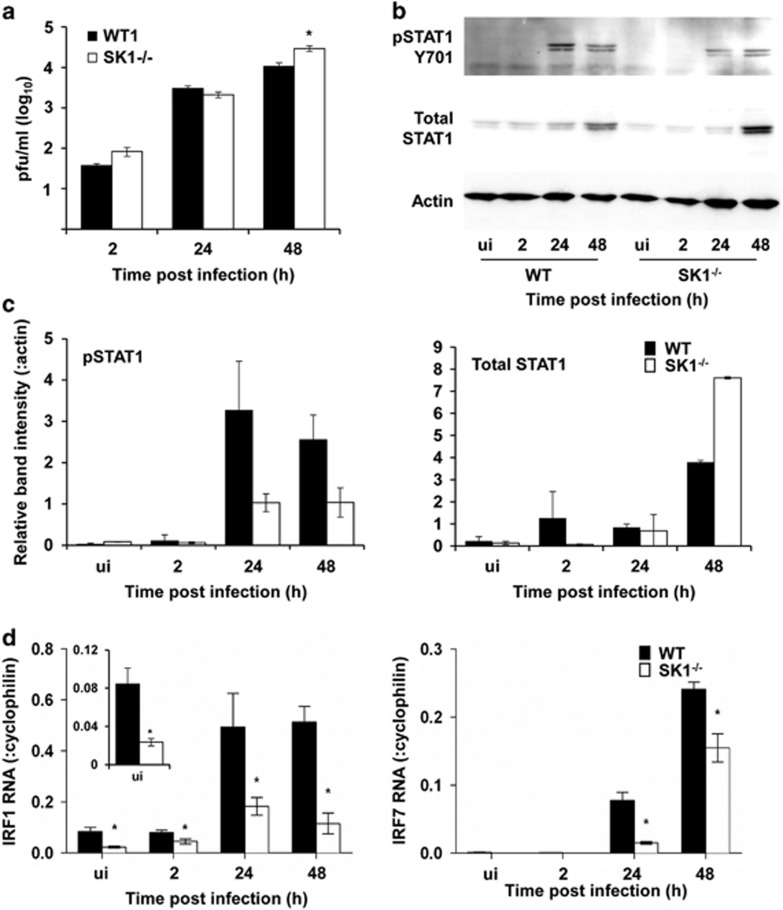
Poor induction of Y701-p-STAT1, IRF1 and IRF7 in SK1^−/−^ pMEF following DENV infection. WT and SK1^−/−^ pMEFs were infected with DENV and at the indicated time point pi culture supernatant, total cellular protein and RNA were harvested. (**a**) infectious virus release was quantitated from cell culture supernatant by plaque assay. Results are shown from *n*=2 WT and SK1^−/−^ pMEF isolates and representative of *n*=6 independent pMEF isolations and infection experiments; (**b**) total protein was subjected to SDS-PAGE and western blot. Filters were serially probed for Y701-pSTAT1, total STAT1 and actin. Bound proteins were detected by chemiluminescence and images collected with a LAS-4000. Results from a single experiment are shown; (**b**) western blots from replicated experiments in two independent pMEF preparations and DENV infections were normalised and quantitated using Image J. Results represent av±s.d., *n*=2. (**c**) Total RNA was extracted and subjected to RT-PCR for IRF1 and IRF7. Results were normalised against cellular cyclophilin and relative RNA level determined by ΔCt method. Data represent av±s.e.m. from *n*=2 RT-PCR values from *n*=3 independent pMEF preparations and infection experiments. *P<0.05, Student's unpaired *t*-test.

**Figure 3:**
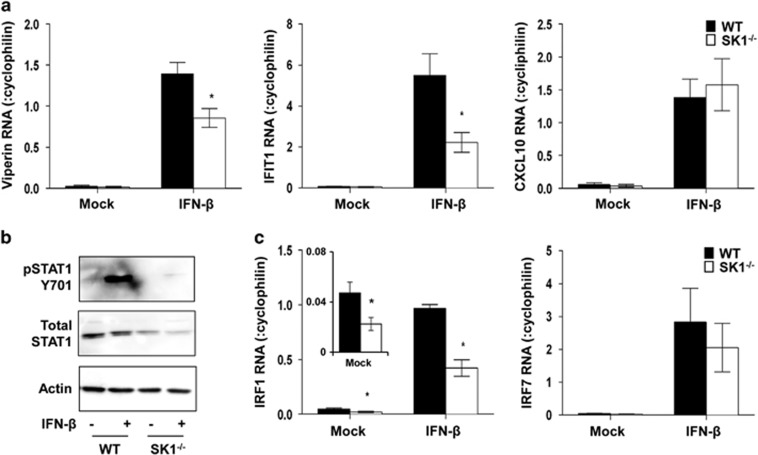
IFN-β induction of IFN-stimulated pathways is reduced in SK1^−/−^ pMEF. WT and SK1^−/−^ pMEFs were stimulated with IFN-β (200 pg/ml). (**a**) At 8 h post stimulation, total RNA was harvested and subjected to RT-PCR for viperin, IFIT1 and CXCL10. Results were normalised against cellular cyclophilin and relative RNA level determined by ΔCt method. Data represent av±s.e.m. from *n*=2 RT-PCR values from *n*=2 independent pMEF preparations and stimulation experiments. *P<0.05, Student's unpaired *t*-test. (**b**) After 15–20 min IFN-β stimulation total protein was harvested and subjected to SDS-PAGE and western blot. Filters were serially probed for Y701-pSTAT1, total STAT1 and actin. Bound proteins were detected by chemiluminescence and images collected with a LAS-4000. Representative western blots are shown from experiments replicated in an independent pMEF preparation (*n*=2). (**c**) Total RNA was extracted and subjected to RT-PCR for IRF1 and IRF7, and results presented are as described in **a**.

A major pathway for type I IFN signalling to induce ISGs is activation of the JAK/STAT pathway, primarily by phosphorylation of STAT1 on tyrosine 701 (Y701; p-STAT1). We next assessed the ability to induce p-STAT1 following DENV infection. Productive DENV infection was observed with 2–3 log increase in infectious virus release compared with input virus at 2 h pi and, as observed previously, a significant increase in virus release from SK1^−/−^ pMEF at 48 h pi (Figure 2a). Concurrent with this increase in infectious virus release, DENV infection induced p-STAT1 in both WT and SK1^−/−^ pMEF, with a reproducible reduction in SK1^−/−^ compared with WT pMEF (Figures 2b and c). Total STAT1 increased at 48 h pi in both WT and SK1^−/−^ cells, as we have previously described in DENV-infected endothelial cells.^29^ Total STAT1 levels tended to be lower in uninfected SK1^−/−^ compared with WT pMEF (see also Figure 3b), although this signal was low and varied between donor MEF preparations. Reliably, total STAT1 levels were increased in SK1^−/−^ compared with WT cells at 48 h pi (Figures 2b and c).

We have previously focused on IFIT1, viperin, CXCL10 and IRF7 mRNA changes following DENV infection,^30^ while there is prior evidence for a linkage of SK1 with IRF1-mediated CXCL10 induction.^26^ Therefore, we defined the changes in IRF1 and IRF7 mRNA in WT and SK1^−/−^ pMEF following DENV infection. Basal levels of IRF1 mRNA were significantly lower in uninfected SK1^−/−^ in comparison with uninfected WT cells, while mRNA levels of IRF7 were very low but not significantly different between the two uninfected cell types (Figure 2d). Following DENV infection, IRF1 and IRF7 mRNA levels increased in both WT and SK1^−/−^ cells. mRNA levels, however, were significantly lower for both IRF1 and IRF7 in SK1^−/−^ compared with WT pMEF (Figure 2d). Together, these results demonstrate similar responses of IFNAR1 protein and IFNAR2 mRNA in WT and SK1^−/−^ pMEF, lower IRF1 in SK1^−/−^ pMEF and a defect in induction of p-STAT1, IRF1 and IRF7 mRNA following DENV infection in SK1^−/−^ pMEF.

### IFN-β-stimulated responses are altered in cells lacking SK1

**Figure 4:**
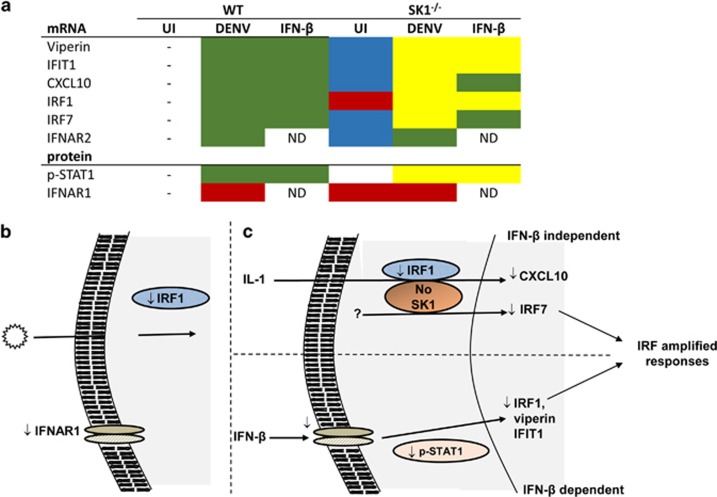
Comparison of changes in protein and mRNAs in WT and SK1^−/−^ pMEF following DENV infection or IFN-β stimulation. (**a**) Relative to WT pMEF: blue, unchanged; green, induced; yellow, induced but significantly lower; red, reduced; ND, not determined; *, inconclusive. (**b**) Summary of changes in basal, uninfected SK1^−/−^ pMEF; (**c**) proposed pathways following DENV infection of SK1^−/−^ pMEF.

We have previously observed that DENV-infected SK1^−/−^ pMEFs produce IFN-β, yet have reduced ability to induce ISG mRNAs,^30^ and, as shown above, a reduced ability to induce p-STAT1 and IRF1 and -7 mRNA. To define the capacity of IFN to directly stimulate responses in SK1^−/−^ pMEF, we treated cells with IFN-β and assessed induction of three key antiviral ISGs, IFIT1, viperin and CXCL10, by RT-PCR, IFNAR signalling via p-STAT1 by western blot as well as IRF1 and IRF7 mRNA by RT-PCR. Mock-treated WT and SK1^−/−^ pMEF had low but similar levels of IFIT1, viperin and CXCL10 mRNA (Figure 3a). As expected, stimulation of cells with IFN-β resulted in increased mRNA levels for all genes in both WT and SK1^−/−^ pMEF. Induction of viperin and IFIT1 mRNA by IFN-β was, however, attenuated in SK1^−/−^ compared with WT cells (Figure 3a). In contrast, stimulation of cells with IFN-β induced comparable levels of CXCL10 mRNA in both WT and SK1^−/−^ cells (Figure 3a). In addition, IFN-β stimulation of cells produced a clear induction of pSTAT1 in WT cells, a response that was markedly reduced in SK1^−/−^ cells (Figure 3b). As suggested in Figures 2b and c, basal levels of total STAT1 appeared lower in SK1^−/−^ compared with WT pMEF (Figure 3b). The total STAT1 signal was low and varied between pMEF preparation and, thus, further quantitation of changes in total STAT1 was not reliable to pursue using western blot. Consistent with results in Figure 2d, IRF1 mRNA is reduced in mock-treated SK1^−/−^ cells (Figure 3c). Further, consistent with viperin and IFIT1 responses, stimulation of cells with IFN-β resulted in poor induction of IRF1 in SK1^−/−^ compared with WT cells (Figure 3c). In contrast, but consistent with CXCL10 responses, stimulation of cells with IFN-β induced comparable levels of IRF7 mRNA in both SK1^−/−^ and WT cells (Figure 3c). This suggests that IFN-β induction of IFIT1, viperin, IRF1 and pSTAT1 protein is influenced by SK1, while IFN-β induction of CXCL10 and IRF7 is SK1-independent. These differential responses to DENV infection and IFN-β stimulation are summarised in Figure 4a.

### SK1^−/−^ pMEFs have normal morphology but reduced levels of cell-surface IFNAR1 and IRF1

**Figure 5:**
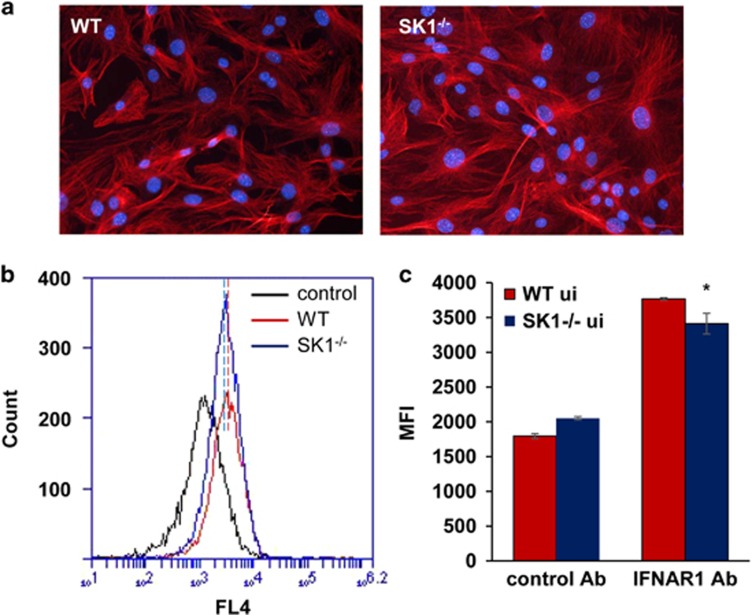
Uninfected SK1^−/−^ pMEFs have lower levels of IFNAR1 but normal cell morphology. (**a**) Uninfected WT and SK1^−/−^ pMEFs were fixed and immunostained with α-tubulin with secondary detection using Alexa-555. Nuclei were stained with Hoechst and images collected by confocal microscopy. Representative images are shown. (**b**) Cells were harvested by scraping and stained using APC-conjugated IFNAR1 or isotype control antibodies, fixed and subjected to flow cytometery. Representative histograms are shown, with the minor change in MFI indicated by a vertical line; (**c**) as in **b** with av±s.e.m. of MFI from replicate flow analysis from *n*=2 independent pMEF preparations. Results from **b**, **c** are from the same experiments shown in Figure 1. Data represent av±s.e.m. from *n*=3 values. *P<0.05, Student's unpaired *t*-test.

As shown above, uninfected SK1^−/−^ pMEFs have reduced IRF1 mRNA compared with WT cells (Figures 2c and 3c), suggesting the potential for adaptive changes in DENV-induced and IFN-stimulated responses in SK1^−/−^ pMEF. We further compared uninfected WT and SK1^−/−^ pMEF. α-Tubulin staining of uninfected cells validated similar cell morphology in WT and SK1^−/−^ pMEF (Figure 5a). Comparison of IFNAR1 on uninfected cells by flow cytometry, as shown in Figure 1, demonstrates a modest but reproducible and significant reduction in cell-surface IFNAR1 in uninfected SK1^−/−^ compared with WT pMEF (Figures 5b and c). These additional changes in uninfected SK1^−/−^ cells are summarised in Figure 4a.

### Acute inhibition of SK1 does not affect components of the IFN-stimulated pathways

**Figure 6:**
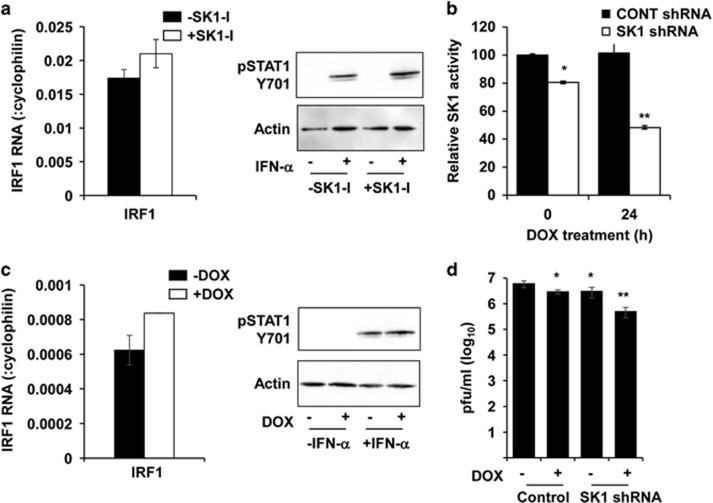
Chemical or shRNA inhibition of SK1 does not affect basal IRF1 mRNA or IFN-β induction of p-STAT1 but SK1 shRNA expression reduces DENV infection. (**a**) HEK293 cells were treated with SK1-I for 90 min, and then stimulated with or without IFN-α (500 U ml^−1^) and total RNA and protein harvested. RNA was subjected to RT-PCR for IRF1 with normalisation against cellular cyclophilin and relative RNA level determined by ΔCt method. Data represent av±s.e.m. from *n*=3 RT-PCR values from *n*=2 independent pMEF replicates. Protein was subjected to SDS-PAGE and western blot. Filters were serially probed for Y701-pSTAT1 and actin. Bound proteins were detected by chemiluminescence and images collected with a LAS-4000. Results are shown from a single experiment that was replicated (*n*=2). (**b**) Control or SK1 shRNA cells were treated with DOX for the time indicated. Cells were harvested, lysed and SK1 activity quantitated. Activity was normalised against total protein and expressed relative to activity in control shRNA cells. Data represent av±s.d. from *n*=2 values. * indicates significantly different to control 0-h post DOX, ** indicates significantly different to control or SK1 shRNA 0-h post DOX, *P*<0.05, Student's *t*-test. (**c**) HEK293 cells containing a DOX-inducible SK1 shRNA were treated overnight with DOX and then stimulated with IFN-α and IRF1 mRNA, and Y701-p-STAT1 measured as described in **a**; (**d**) HEK293 cells containing a DOX-inducible control or SK1 shRNA were treated overnight with DOX, DENV-infected and supernatants harvested at 24 h pi and analysed by plaque assay. Results represent av±s.e.m. from *n*=4 assay values from *n*=2 independent infection experiments. * indicates significantly different to control-DOX, ** indicates significantly different to SK1 shRNA-DOX, *P*<0.05, Student's *t*-test.

Our prior studies have demonstrated reduced DENV induction of ISGs in HEK293 cells treated with the SK1-I.^30^ This, however, is accompanied by a reduced DENV infection, and thus this system is not useful to discriminate effects on ISGs that are due to reduced SK1 activity compared with reduced induction by DENV replication *per se*. We thus assessed whether the adaptive changes observed above in uninfected SK1^−/−^ pMEF (reduced IRF1 mRNA and ability to induce p-STAT1) that are associated with the phenotype of enhanced DENV infection and reduced IFN-stimulated responses were paralleled by a chemical reduction in SK1. HEK293 cells were pretreated for 90 min with SK1-I, RNA extracted and quantitated by RT-PCR. Results show no change in basal levels of IRF1 mRNA (Figure 6a). Further, IFN-α stimulation of SK1-I-treated HEK293 showed no major difference in the ability to induce p-STAT1 (Figure 6a). As an alternative method to reduce both SK1 activity and protein in human cells, we generated HEK293 cells with doxycycline (DOX)-inducible expression of a SK1 shRNA that is linked to red florescent protein expression. SK1 activity in these cells is significantly reduced by ~20%, likely due to leakiness of the promoter driving the SK1 shRNA (Figure 6b). In addition, overnight DOX treatment of these cells induced red florescent protein and a further significant reduction in SK1 activity (Figure 6b). RNA was extracted from uninfected cells and subjected to RT-PCR for IRF1. Consistent with results of SK1-I treatment, SK1 shRNA expression did not induce any change in basal levels of IRF1 mRNA (Figure 6c). In addition, SK1 shRNA-expressing cells were stimulated with IFN-α for 20 min, cells lysed and pSTAT1 analysed by western blot. Results demonstrate no difference in ability of IFN-α to induce p-STAT1 in cells induced to express the SK1 shRNA (Figure 6c). shRNA-expressing cells were also DENV-infected and at 24 h pi supernatant collected and analysed for infectious virus release by plaque assay. Of note, DOX treatment of control cells, or non-induced SK1 shRNA cells showed reduced DENV infection. This is further significantly reduced, approximately one log by DOX treatment in SK1 shRNA cells (Figure 6d). Thus, a chemical or shRNA-mediated reduction in SK1 is not sufficient to reproduce the adaptive changes in basal components of the IFN and pathogen recognition responses that we observe in SK1^−/−^ cells. Further, in contrast to the enhanced infection seen in SK1^−/−^ pMEF but consistent with SK1-I and SKi treatment of cells,^30^ expression of SK1 shRNA is associated with reduced DENV infection.

## Discussion

Following viral infection of a cell, there is a complex host antiviral response, including directly stimulated responses and secondary IFN-stimulated pathways. Here we have defined novel innate responses following DENV infection, including upregulation of mRNA for IRF1 and -7, upregulation of mRNA for IFNAR2 and downregulation of cell-surface IFNAR1. Our data also show that a genetic lack of SK1 can influence specific aspects of responses to infection and type I IFN stimulation, extending the evidence that implicates SK1 in the cellular response to DENV.^20, 30, 31^

Our prior data have suggested poor induction of ISGs following DENV infection in SK1^−/−^ pMEF.^30^ We further show that following IFN-β stimulation, viperin, IFIT1, p-STAT1 and IRF1 but not CXCL10 or IRF7 induction is compromised in the absence of SK1. This suggests a role for SK1 in type I IFN-stimulated JAK/STAT-mediated activation of some ISGs, but additionally the involvement of a SK1-dependent pathway *not* involving the JAK/STAT pathway for induction of IRF7 and CXCL10 that is activated during a DENV infection. On the basis of literature, we suggest that this pathway may be via interleukin-1, known to be induced during DENV and responsible for induction of CXCL10, via a pathway described to be positively regulated by SK1.^26^ This is summarised in Figure 4b. In contrast, the ability of IFN to induce p-STAT1 was not reduced in cells acutely treated with an inhibitor of SK1 activity (SK1-I) or expressing a SK1 shRNA. A longer treatment of cells with SK1-I was not employed as this can reduce cell proliferation, which will confound analysis. Given these caveats, we suggest that the defect in DENV and IFN-β-stimulated responses in SK1^−/−^ pMEF reflects a biological link of the SK1 and type I IFN pathway that is likely mediated by secondary adaptive responses to long-term loss of SK1 and disruption of the SK/S1P homeostatic balance.

In support of this we demonstrate alterations in uninfected SK1^−/−^ cells that could influence type I IFN responses. SK1^−/−^ pMEFs have normal α-tubulin staining, demonstrating that the poor DENV and IFN-β responses in SK1^−/−^ cells are not due to gross morphological abnormalities that may affect multiple signalling pathways. In fact, in contrast to the poor induction of some ISGs in SK1^−/−^ cells, mRNA for IFNAR2 is induced normally and IFNAR1 protein undergoes downregulation in response to DENV infection in a manner comparable to WT cells. This demonstrates that many of the DENV-induced responses are normal in SK1^−/−^ pMEF. The downregulation of IFNAR1 protein we have demonstrated here following DENV infection has been previously reported for other flaviviruses^32^ and shown to be due to NS5 antagonism of the host cell prolidase protein, which is involved in transport of IFNAR1 to the cell surface.^33^ Stimulation of cells with S1P, the product of SK1 action, reportedly leads to internalisation of S1PR1, co-internalisation of IFNAR1 into endosomes and refractory responses to IFN-α.^34^ In addition to DENV-mediated downregulation of IFNAR1, we have also observed a small but significant reduction in the basal levels of IFNAR1 in uninfected SK1^−/−^ cells. We thus propose that S1PR1-mediated downregulation of IFNAR1 may be a mechanism underlying the lower IFNAR1 and contributing to refractory responses of SK1^−/−^ cells to DENV and IFN-β seen in our study.

Since prior data from our laboratory demonstrated poor induction of IRF7 in the absence of SK1 and elevated basal IRF7 in SK1^−/−^ immortalised MEF,^30^ in combination with the prior literature linking CXCL10, IRF1 and SK1,^26^ we rationalised to assess IRF1 and -7 and we have shown that DENV infection induces both IRF1 and -7 mRNA. IRF7 is well described to be induced by RNA viruses and toll-like receptor-3 stimulation, and we previously reported significant upregulation of IRF7 in DENV-infected MEF by microarray analysis.^30^ IRF1 is traditionally described to be induced by cGAS activation in response to DNA viruses, and our observed induction of IRF1 during DENV infection supports the growing data demonstrating the importance of cGAS-stimulated gene expression during RNA virus infections.^5, 35, 36^ Further, our data demonstrate decreased basal IRF1 mRNA in SK1^−/−^ pMEF but not following chemical or shRNA inhibition of SK1. This difference may relate to the reduced ability to affect the total cellular pool of SK1 protein or IRF1 mRNA in the shorter time frame of chemical or shRNA inhibition of SK1. SK1 has role in ubiquitination of IRF1^26^ and may stabilise basal protein levels, which with long-term lack of SK1 protein and activity (as in SK1^−/−^ cells) but not acute reduction in SK1 (as in SK1-I or SK1 shRNA treatment) could potentially feedback to change basal levels of IRF1. While the SK1 dependency of IRF1 induction during DENV infection was reflected by IFN-β stimulation, the SK1-dependent induction of IRF7 seen following DENV infection was not. Similar to our arguments for CXCL10, this implies a SK1-dependent response during DENV infection that is not via IFN-β and acts to induce IRF7 as summarised in Figure 4b. Regulation of IRF7 transcription is reported to be primarily driven by the type I IFNs^37^ but IRF7 has also been described to be induced following viral infection, such as Sendai virus, by IFN-β-independent direct viral-stimulated pathways.^38^ Potentially, SK1 may influence these direct IFN-β-independent pathways, whereby RNA viruses stimulate IRF7.

IRF1 and -7 are ‘master regulators’ of the IFN response and are involved in a positive feedback loop that regulates the transcription of IFNs. We focused on IRFs and analysed predicted promoter elements that could potentially have a role in discriminating the SK1 dependency of responses of viperin, IFIT1 and CXCL10 (Table 1). All gene promoters contained several common response elements and at least one predicted element for IRF and NFκB. Viperin and IFIT1 both contain multiple IFN-stimulated response elements, while CXCL10 did not, and thus this element correlates with SK1 and IFN-β dependency. IFIT1 has a total of five, and CXCL10 a total of three different IRF elements. IFIT1 and viperin contain an IRF7 promoter element, while only IFIT1 and CXCL10 have an IRF1 element (Table 1). However, type I IFN-independent induction of viperin, that is driven by IRF1, has previously been described in the context of Newcastle disease virus and vesicular stomatitis virus infection.^39^ As viperin, IFIT1 and CXCL10 have promoter elements for IRF1 and/or IRF7, it is likely that lower levels of these IRFs will contribute to the later poor induction of these ISGs in the absence of SK1. Dissecting the mechanisms by which SK1, IRF1 and IRF7 contribute to transcription of viperin, IFIT1 and CXCL10 requires further experimentation, but it is clear that functional deficits in SK1 that has an impact on IRF induction will have major consequences for the cellular IFN response to infection (Figure 4b). In addition, the described role for SK1 in stimulating NFκB-driven gene expression would be predicted to have a major impact on induction of CXCL10, which contains four NFκB elements, following DENV infection but not IFN-β stimulation, and this is consistent with our observations.

A key finding in our study is reduced p-STAT1, an indicator of activation of the JAK/STAT pathway, in DENV-infected or IFN-β-treated SK1^−/−^ pMEF. S1P has been previously implicated in the JAK/STAT pathway, where a reduction in S1P through overexpression of S1P lyase resulted in elevated p-STAT1 and inhibition of IAV infection.^28^ Conversely, IAV infection of cells overexpressing SK1 leads to reduced p-STAT1 and enhanced IAV infection.^28^ Our experimental system differs with respect to the IAV infection model of Seo *et al.*,^28^ with different viral stimuli and the added complexity of DENV-mediated downregulation of IFNAR1. Both experimental systems represent extremes of SK1 activity with ~500-fold overexpression of SK1^28, 40^ compared with our genetic lack of SK1. The SK1/S1P axis is subject to strong autoregulation,^41^ and both the findings of Seo *et al.*,^28^ and our contrasting results, showing reduced type 1 IFN responses with high and low SK1 levels, respectively, could be reconciled by S1PR1-mediated downregulation of IFNAR1^34^ leading to reduced ability to induce p-STAT1. Our demonstration of a small but significant reduction in IFNAR1 in SK1^−/−^ cells shows that this does occur in our system, although the molecular pathways by which the lack of SK1 leads to reduction in S1PR1 and downregulation of IFNAR1 in SK1^−/−^ pMEF remain to be defined. Further, we have also demonstrated reduced basal IRF1 in SK1^−/−^ pMEF. Together, these changes in IFNAR1 and IRF1 would be predicted to place SK1^−/−^ cells in a basal state where pathogen recognition and stimulation of IFNAR signalling, leading to p-STAT1 and transcription of ISGs, may be compromised (Figure 4b). These effects of SK1^−/−^ on IRF1 and p-STAT1 are not observed in cells chemically treated or expressing a shRNA to induce a short-term reduction in SK1, supporting the idea that these are adaptive changes as a result of chronic reduction in SK1. Overall, these adaptive changes and subsequent defects in DENV-induced and IFN-β-stimulated pathways in SK1^−/−^ pMEF correlate with our previously defined enhanced DENV infection. In contrast, SK1-I or SK1 shRNA treatment that is not associated with these adaptive changes is associated with inhibition of DENV infection, reconciling the differences in DENV susceptibility seen in these different systems for manipulating SK1.

In summary, our results implicate SK1 as a new factor that can influence the susceptibility to DENV infection and induction of antiviral and type I IFN-stimulated responses. The effect of SK1 on these cellular functions is dependent on the context of the changes in SK1, which is likely related to the close homeostatic regulation of the SK/S1P axis. In some contexts the reduction in SK1 can be antiviral without loss of innate responses to infection. This suggests the potential to discriminate antiviral and IFN-stimulated response by changes in the SK1/S1P axis, which, given the growing availability of pharmaceuticals to modulate the SK1/S1P axis,^42, 43^ becomes an attractive option to consider. The contrasts in our outcomes between different systems for manipulating SK1 however highlight the complexity of these interactions and herald caution in extrapolating outcomes from genetic lack of SK1 to effects of chemical or shRNA-mediated inhibition of SK1.

## Materials and methods

### Cell culture

pMEFs were generated from six different WT and SK1^−/−^ embryos and frozen stocks generated at passage 3. Mice were used in accordance with animal ethics 181/12 approved by the SA Pathology/CALHN animal ethics committee. All MEF cultures were used in infection studies within 2 weeks of thawing. Individual studies were verified in at least two different pMEF cultures. Cells were treated in culture for 15–20 min with recombinant mouse IFN-β (Biolegend, San Diego, CA, USA) or pretreated for 90 min with 5 μM SK1-I (Enzo Life Sciences, Farmingdale, NY, USA) prior to stimulation with IFN-β. HEK293 cells expressing control or SK1 shRNA were generated following lentivirus transduction and selection as previously described.^44^ Cells were passaged in tetracycline-free media (Dulbecco's modified Eagles medium (DMEM) with 10% (v/v) fetal calf serum) to minimise basal shRNA expression.

### DENV infections and plaque assay

Cells were infected at a multiplicity of infection of 1 for 90 min and utilised Mon601, a full-length DNA clone of the DENV type-2 (DENV-2) strain New Guinea C,^45^ produced as described previously.^46^

### Reverse transcription real-time polymerase chain reaction

Total RNA was extracted from cells using Trizol (Gibco, Gaithersburg, MD, USA) DNase-treated and subjected to reverse transcription (RT) using random hexamers (NEB, Ipswich, MA, USA). Complementary DNA was subjected to real-time polymerase chain reaction (PCR) using primers and quantitation methods as described previously.^30^ Additional primers, not described in Clarke *et al.,*^30^ were used for mouse (m)-IFNAR2 (forward [f] GGATGGCAGTGACAGTGAC; reverse [r] ATGGAGAACCCTCAGAAACAC), m-IRF1 (f-CAGAGGAAAGAGAGAAAGTCC; r-CACACGGTGACAGTGCTGG). RT-PCR included *n*=2 assay replicates and *n*=2–3 biological repeats in independent pMEF isolates. Reactions included internal standards; results were normalised against cylcophilin and relative RNA level determined by ΔCt method.

### Sodium dodecyl sulphate-polyacrylamide gel electrophoresis and western blotting

Cells were lysed in extraction buffer (50 mM Tris pH 7.4, 150 mM NaCl, 2 mM Na_3_VO_4_, 10 mM NaF, 1 mM EDTA, 10% (v/v) glycerol, 0.05% (v/v) triton X-100, 10 mM beta-glycerophosphate, 1 mM dithiothreitol) with protease inhibitors (Roche Complete Mini, Roche, Hawthorn, VIC, Australia) and protein levels quantitated (Bio-Rad protein assay, Gladesville, NSW, Australia). Thirty micrograms of total protein were subjected to sodium dodecyl sulphate-polyacrylamide gel electrophoresis, proteins transferred to nitrocellulose membranes and membranes serially incubated with antibodies for Y701 phosphorylated STAT1 (cat #9167, Cell Signaling Technology, Danvers, MA, USA), total STAT1 (cat #9172, Cell Signaling Technology) and actin (anti actin, clone C4 cat #MAB1501R, Millipore, Temecula, CA, USA). Bound antibody complexes were detected by horseradish peroxidase-conjugated secondary antibodies with detection by chemiluminescence (Clarity ECL substrate, Bio-Rad) and collection of image with LAS-4000. Images were quantitated using Image J (https://imagej.nih.gov/ij/).

### Immunofluorescent staining for α-tubulin

Uninfected WT and SK1^−/−^ pMEF were seeded on gelatin-coated glass coverslips, cultured and fixed in 4% (w/v) paraformaldehyde in phosphate-buffered saline. Fixed cells were washed, permeabilised in 0.05% (v/v) IGEPAL (Sigma, St Louis, MO, USA) in phosphate-buffered saline, stained with α-tubulin with detection using a secondary anti-mouse IgG-Alexa-555 (Molecular Probes, Eugene, OR, USA) and nuclear staining with 5 μg ml^−1^ Hoechst (Molecular Probes). Images were captured by confocal fluorescent microscopy (Leica SP5, Nussloch, Germany). Immunostaining was repeated on *n*=2 independent pMEF isolations.

### Flow analysis of IFNAR1

Cells were cultured, left uninfected or DENV-infected, as described above. At 24 h post infection, cells were harvested, blocked in phosphate-buffered saline with 3% (w/v) bovine serum albumin and immunostained with control IgG (APC Mouse IgG1, cat 3400119, BioLegend) or anti-IFNAR1 (allophycocyanin (APC) anti-mouse IFNAR1, cat #127313, BioLegend)-conjugated antibody. Stained cells were fixed and subjected to flow cytometry (Accuri, BD systems, Franklin Lakes, NJ, USA). Staining was performed in triplicate and repeated on *n*=2 independent pMEF isolations.

### SK1 activity assays

Cells were lysed in extraction buffer and total protein quantitated, as described above for sodium dodecyl sulphate-polyacrylamide gel electrophoresis analysis. SK1 activity assay was quantitated by ^32^P transfer to sphingosine *in vitro*, as described previously.^47^

### Promoter analysis of IFIT, viperin and CXCL10

Promoter sequences 1000 bp upstream, and 50 bp downstream from the transcription start site, were obtained for each gene using Promoser. Potential transcription factor-binding sites were identified using MatInspector (Genomatix Suite, Muchen, Germany).

### Statistics and data analysis

All experiments were performed at a minimum of duplicate samples with a minimum of *n*=2 biological replicates or as stated in figure legends. Statistical differences were determined by Student's unpaired *t*-test with a *P*<0.05.

## Figures and Tables

**Table 1:** Promoter analysis of major antiviral response elements in human *viperin*, *IFIT1* and *CXCL10* genes was performed by identification of promoter regions with ‘Promoser’ and potential transcription factor-binding sites using MatInspector (Genomatix Suite)

*Promoter element; Gene name*	*ISRE*	*IRF*					*NFkB*
		*1*	*2*	*3*	*7*	*8*	
Viperin	2	–	–	–	1	–	1
IFIT1	3	1	2	1	1	–	1
CXCL10	–	1	1	–	–	1	4

Abbreviations: IRF, interferon regulatory factor; ISRE, interferon-stimulated response element; NFkB, nuclear factor κB.
